# Alanine Aminotransferase and Risk of the Metabolic Syndrome: A Linear Dose-Response Relationship

**DOI:** 10.1371/journal.pone.0096068

**Published:** 2014-04-29

**Authors:** Setor K. Kunutsor, Dorothy Seddoh

**Affiliations:** 1 Department of Public Health and Primary Care, University of Cambridge, Strangeways Research Laboratory, Cambridge, United Kingdom; 2 Maranatha University College, Accra, Ghana; University Heart Center Freiburg, Germany

## Abstract

**Background:**

Elevated baseline circulating alanine aminotransferase (ALT) level has been demonstrated to be associated with an increased risk of the metabolic syndrome (MetS), but the nature of the dose-response relationship is uncertain.

**Methods:**

We performed a systematic review and meta-analysis of published prospective cohort studies to characterize in detail the nature of the dose-response relationship between baseline ALT level and risk of incident MetS in the general population. Relevant studies were identified in a literature search of MEDLINE, EMBASE, and Web of Science up to December 2013. Prospective studies in which investigators reported relative risks (RRs) of MetS for 3 or more categories of ALT levels were eligible. A potential nonlinear relationship between ALT levels and MetS was examined using restricted cubic splines.

**Results:**

Of the 489 studies reviewed, relevant data were available on 29,815 non-overlapping participants comprising 2,125 incident MetS events from five prospective cohort studies. There was evidence of a linear association (*P* for nonlinearity = 0.38) between ALT level and risk of MetS, characterised by a graded increase in MetS risk at ALT levels 6–40 U/L. The risk of MetS increased by 14% for every 5 U/L increment in circulating ALT level (95% CI: 12–17%). Evidence was lacking of heterogeneity and publication bias among the contributing studies.

**Conclusions:**

Baseline ALT level is associated with risk of the MetS in a linear dose-response manner. Studies are needed to determine whether the association represents a causal relationship.

## Introduction

Metabolic syndrome (MetS) is characterized by a constellation of disorders, including high blood pressure, dyslipidemia, hyperglycemia, and abdominal obesity, and has been consistently shown to be strongly associated with type 2 diabetes[1], [2] and cardiovascular disease.[1], [3] The Third Report of the National Cholesterol Education Program – Adult Treatment Panel (NCEP-ATP) III has stressed the importance of targeted preventive approaches for individuals with the MetS.[4] Nonalcoholic fatty liver disease (NAFLD), commonly regarded as the hepatic component of the metabolic syndrome,[5], [6] is a clinical condition characterised by hepatic steatosis with varying degrees of necroinflammation and fibrosis, and which develops in the absence of substantial alcohol intake.[5] Reports indicate that NAFLD is the common cause of chronically and unexplained elevated levels of liver enzymes, particularly the aminotransferases.[7], [8] Serum alanine aminotransferase (ALT) is the liver enzyme most strongly correlated with liver fat accumulation[9] and elevated ALT levels are almost commonly used to identify NAFLD.[10], [11].

There has been considerable uncertainty regarding the prospective association between ALT level and the MetS until recently. In a review published in a recent issue of PLoS ONE, Liu and colleagues[12] synthesized available prospective epidemiological data on the association between ALT and incident MetS and reported a multivariate adjusted relative risk (RR) (95% confidence interval) of 1.81 (1.49–2.14) for MetS in a comparison of top versus bottom category of baseline ALT level. The risk for MetS was 1.13 (1.11–1.16) per 5 U/L increment in ALT level in dose-response analysis. Detailed characterization of the nature of the dose-response relationship is however still lacking, as this was not addressed by previous studies and the recent review. It is not clear if there is a continuous dose-response relationship to the association across the whole range of ALT levels or there is a threshold effect. It is important to establish this, especially if there exists a threshold which would potentially optimize the detection of individuals at increased risk of the MetS. To help clarify the evidence, we report an updated analysis which aims to quantify and characterize in detail the nature of the dose-response relationship between ALT level and risk of MetS.

## Methods

This review was conducted using a predefined protocol and reported in accordance with PRISMA guidelines (**Checklist S1**).[13] We searched MEDLINE, EMBASE, and Web of Science electronic databases up to December 2013, for prospective (cohort, case-cohort or “nested case control”) population-based studies reporting on the associations between ALT level and MetS risk. The computer-based searches combined free and MeSH search terms and combination of key words related to ALT (e.g., “alanine aminotransferase”) and MetS (e.g., “metabolic syndrome”). There were no restrictions on language or the publication date. Reference lists of retrieved articles were manually scanned for all relevant additional studies and review articles. We restricted the search to studies of humans. Observational cohort studies were included if they had at least one year of follow-up, assessed associations of ALT with incident MetS in adults, with samples measured at baseline, and recruited participants from approximately general populations (i.e., they did not select participants on the basis of confirmed pre-existing medical conditions such as MetS, diabetes mellitus, or known liver diseases at baseline). Studies which reported RRs with 95% confidence intervals (CIs) for at least three quantitative ALT categories were included. The RR with 95% CIs was used as the common measure of association across studies. We used generalized least-squares trend estimation (GLST) analysis as described by Greenland and Orsini[14], [15] to compute the trend from the correlated natural logs of the RRs across categories of ALT. For studies that presented results separately according to subgroups (e.g., by sex), separate dose-response trends were derived and a within-study summary estimate was obtained using a fixed effect analysis. The dose-response trends are presented for a 5 U/L increment in ALT level. We examined a potential nonlinear dose-response relationship between ALT levels and MetS by modeling ALT levels using restricted cubic spline functions with 3 knots at percentiles 25%, 50%, and 75% of the distribution.[16] A *P* value for nonlinearity was calculated by testing the null hypothesis that the coefficient of the second spline is equal to zero. Study-specific results were combined using random-effects models. Consistency of findings across studies was assessed by standard *χ* ^2^ and *I* ^2^ statistics, with I^2^>50% considered to be important.[17], [18] Evidence of publication bias was assessed using Begg’s funnel plots and Egger’s asymmetry test.[19], [20] All analyses were performed using Stata release 12 (StataCorp, College Station, Texas).

## Results

### Study Selection and Characteristics

**Figure 1:**
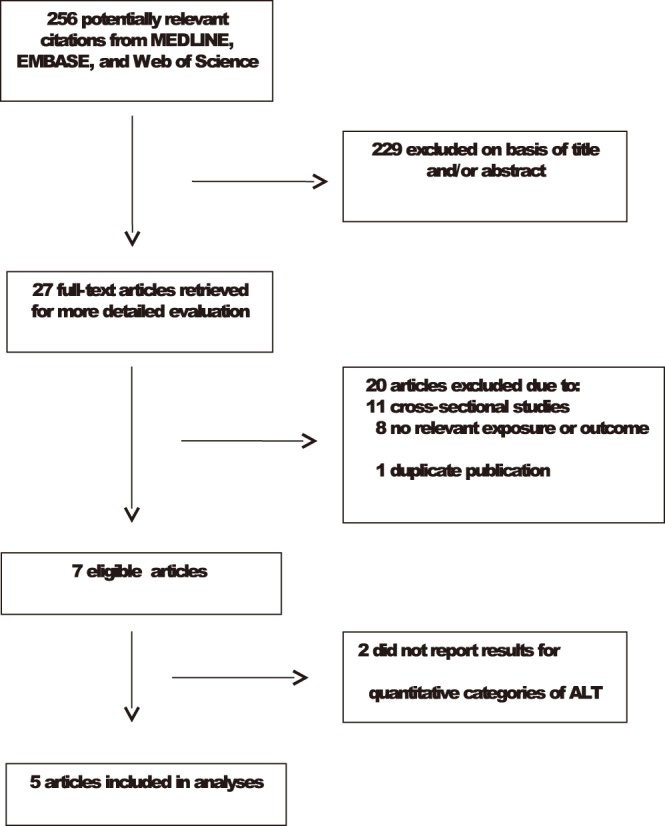
Selection of studies included in the meta-analysis. ALT, alanine aminotransferase.

Our initial search identified 489 potentially relevant citations. Following initial screening and detailed assessments, seven studies were potentially eligible (**Figure 1**). Of the seven eligible prospective cohort studies,[21]–[27] relevant data were available on 29,815 nonoverlapping participants from five studies carried out in Europe (France and the Netherlands) and Asia (Japan, Korea, and China). The cumulative analysis involved 2,125 incident MetS events, collected over average follow-up periods ranging from 3 to 7 years. Three studies ascertained the diagnosis of MetS according to the previous NCEP-ATP III criteria, defined as the presence of three or more of the following components: fasting glucose≥6.1 mmol/L (110 mg/dL), high-density lipoprotein (HDL) cholesterol<1.0 mmol/L (40 mg/dL) in men or<1.3 mmol/L (50 mg/dL) in women, triglycerides≥1.7 mmol/L (150 mg/dL), waist circumference≥102 cm in men or≥88 cm in women, and blood pressure≥130/85 mmHg.[4] Of the three, one study used a cut-off for waist circumference that was more appropriate for an Asian population.[27] Two studies used the International Diabetes Federation criteria (IDF), defined as waist circumference with ethnic specific cut-offs for men and women plus any two of the following: triglycerides>1.7 mmol/L (150 mg/dL), HDL cholesterol<1.03 mmol/L (40 mg/dL) in men or 1.29 mmol/L (50 mg/dL) in women or specific treatment for this lipid abnormality, blood pressure≥130/85 mmHg or treatment of previously diagnosed hypertension, and fasting plasma glucose≥5.6 mmol/L (100 mg/dL) or previously diagnosed type 2 diabetes[28] (**Table 1**). Two studies substituted body mass index (BMI) for waist circumference in their definition of abdominal obesity.[25], [26].

**Table 1:** Characteristics of published prospective studies contributing to dose-response analysis of alanine aminotransferase and risk of metabolic syndrome.

Lead author, publication year	Name of study or source of participants	Locationofstudy	Year(s)of baseline survey	Baseline age range(years)	% male	Durationoffollow-up	Total no.of participants	No.ofcases	MetS casedefinition	Covariates adjusted for
Andre et al., 2007	DESIR	France	1994–1996	30–65	46.7	3	3,545	309	IDF criteria	Age and GGT
Schindhelm et al., 2007	Hoorn	Netherlands	1989	50–75	42.4	6	1,097	226	NCEP-ATP III criteria	Age, sex, alcohol intake, follow-up duration, waist, glucose, HDL-C, triglycerides, systolic and diastolic BP
Jo et al., 2009	HPC	Korea	2002	19–86	70.8	4	21,535	802	IDF criteria	age and GGT
Nakanishi et al., 2004	Office Workers	Japan	1994	35–59	100	7	2,957	608	NCEP-ATP III criteria	Age, family history of diabetes, BMI, alcohol intake, cigarette smoking, regular physical activity, WBC count, other liver markers
Xu et al., 2011	City of Shangai	China	2004–2008	>/ = 40	60.2	4	681	180	modified NCEP-ATP III criteria	Age, sex, occupation, educational level, family history of diabetes, smoking, drinking status,leisure-time activity, BMI, HOMA-IR, GGT
*Total*							*29,815*	*2,125*		

DESIR, Data from Epidemiological Study on the Insulin Resistance Syndrome; Health Promotion; HPC, Health Promotion Centre.

BMI, body mass index; BP, blood pressure; GGT, gamma glutamyltransferase; HDL-C, high-density lipoprotein cholesterol; HOMA-IR, homeostasis model assessment of insulin resistance; IDF, International Diabetes Federation; MetS, metabolic syndrome; NCEP-ATP III, National Cholesterol Education Program-Adult Treatment Panel; WBC, white blood cell.

### Alanine Aminotransferase and Risk of Mets

**Figure 2:**
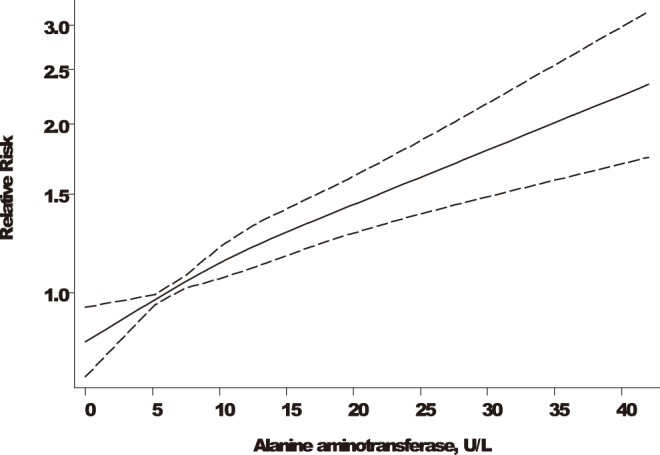
Dose-response relationship between alanine aminotransferase level and relative risk of the metabolic syndrome. Adjusted relative risks and 95% confidence intervals (CIs; dashed lines) are reported. ALT levels were modeled with restricted cubic splines with 3 knots in a random-effects dose-response model. The median value (6 U/L) of the lowest reference range was used to estimate all relative risks. The vertical axis is on a log scale; ALT, alanine aminotransferase.

There was evidence of a linear positive association (*P* for nonlinearity = 0.38) between ALT level and risk of MetS, which was present across the range of ALT values (6–40 U/L) (**Figure 2**). The combined RR (95% CI) of MetS for a 5 U/L increment in ALT level was 1.14 (1.12–1.17). The summary RR was not sensitive to choice of fixed or random effects models and there was no evidence of heterogeneity among the findings of the contributing studies (I^2^ = 0%, 95% CI: 0, 79%; *P* = 0.59). Exclusion of any single study at a time had minimal effect on the pooled RRs, which ranged from 1.11 (1.06–1.16) to 1.15 (1.12–1.18). There was no evidence of publication bias (Egger’s test *P* = 0.12), consistent with observed funnel plot symmetry.

## Discussion

We have confirmed the positive independent association between ALT level and incident MetS risk and also characterized in greater detail the nature of the dose-response relationship. A monotonous dose-response relationship between ALT level and MetS risk was demonstrated, which is present across the range of ALT values (6–40 U/L) without any threshold effect. There are indications that the risk for cardiometabolic disorders including the MetS, seem to increase significantly at levels that are substantially lower than the upper normal limit of ALT. At present, there is no standard level of elevation that is considered abnormal for the liver enzymes including ALT, which is considered a more sensitive indicator of liver injury. However, the established upper normal limit of ALT has been set at an average of 40 U/L ranging from 30–50 U/L over several decades ago.[29] Recent reports from studies conducted in both Asian and Western populations, have suggested that the current reference ranges of ALT level do underestimate the frequency of chronic liver disease and have made recommendations for the revision of the upper limit of normal to be lowered.[30]–[32] Mild and unexplained elevations in ALT levels are very common in the general population and most often than not indicate the presence of subclinical liver disease, most commonly non-alcoholic fatty liver disease. Prati and colleagues[30] redefined the upper normal limits of ALT to 30 U/L for men and 19 U/L for women and have recommended its wide adoption as these levels will improve the sensitivity of ALT for detection of chronic liver disease. Assays for ALT are sensitive, well standardised, simple and inexpensive, and unlike other liver enzymes, are very specific for the liver. It is likely that adoption of these recommendations may help improve the identification of individuals with subclinical liver disease and at risk of developing the MetS and other cardiometabolic conditions.

The overall findings suggest the possibility of a causal relationship, but establishing this requires robust evidence from clinical trials. Randomized controlled trials (RCTs) of pharmacologic agents or interventions that modify levels of ALT and reverse MetS risk, provide the highest level of evidence for establishing whether ALT is causal in MetS. Several interventions are available that influence levels of ALT, however, such available interventions influence levels of several hepatic and lipid factors,[33], [34] making it difficult to disentangle any potential associations attributed to changes in levels of ALT alone. In the absence of such RCTs however, integrative studies of genetic variants specifically related to ALT levels may provide another route to help judge whether ALT is directly causal in MetS (i.e., “Mendelian randomisation [MR] analysis”[35]). A substantial proportion of the variance of ALT is explained by genetic factors, with heritability estimates reported to range between 22 to 48%.[36]–[38] Significant allelic associations with ALT have been reported for several genetic variants in genome-wide association studies (GWAS),[39]–[41] however multiple associations with several cardiometabolic traits have also been observed for majority of the identified loci. In addition, the population variations in circulating levels of ALT accounted for by these variants are very low, ranging from 0.20 to 0.36%.[39]–[41] Larger more adequately powered GWAS may help unravel new variants with larger effects on ALT levels, enabling assessments of any causal association of ALT levels with risk of MetS.

The strengths and potential limitations of this analysis deserve mention. Given that NAFLD represents the hepatic component of the MetS;[5], [6] to be able to demonstrate the ALT-MetS relationship robustly with minimal bias, studies should sufficiently have long follow-up durations and steps should be taken to ensure that participants do not have prevalent NAFLD at baseline or other causes of elevated baseline levels of ALT should be excluded. Our meta-analysis included only studies that had recruited participants from approximately general populations, studies with long follow-up durations (3–7 years), and those that excluded participants with marked elevations in ALT levels, therefore minimising any effects of reverse causation. There was no evidence of heterogeneity or publication bias among contributing studies. Sensitivity testing excluding a single study at a time yielded comparable results, indicating the robustness of the findings. We were unable to fully examine the impact of adjustment for all known and potential risk factors and also combine models in studies that adjusted for the same set of confounders, because of the varying degree of confounder adjustment across individual studies. It was not possible to achieve a comparable outcome definition of MetS across all studies, as different criteria (NCEP-ATP III or IDF) were used and two studies substituted BMI for waist circumference in their definition of abdominal obesity. However, the NCEP-ATP III and IDF definitions have been found to show good agreement in the diagnosis of MetS.[42], [43] Additionally, BMI has been suggested as equally effective as waist circumference for predicting the development of metabolic disorders[44], [45] and has been adopted in previous studies of the MetS.[1], [46] We were also unable to correct the estimates for within-individual variation in levels of ALT over time which may have underestimated the associations, because data involving repeat measurements were not reported by the contributing studies. The eligible studies were mainly carried out in Asian and European populations, which hamper the generalisation of our findings. Reference levels for ALT may vary in different populations, therefore further studies are needed in other geographical locations such as North America and in the Africa Region to investigate these findings.

In conclusion, available evidence suggests a positive independent association of baseline ALT level with risk of the MetS, consistent with a linear dose-response relationship. Further work is required to establish the causality of the association. In the absence of such data however, mild or subtle elevations of ALT levels in individuals should be a cue for further clinical evaluation.

## Supporting Information

Checklist S1 **PRISMA checklist.**
(DOC)
